# Social isolation affects intra‐specific interaction behaviour and reduces the size of the cerebellar brain region in juvenile Atlantic salmon *Salmo salar*

**DOI:** 10.1111/jfb.15142

**Published:** 2022-07-13

**Authors:** Haoyu Guo, Joacim Näslund, Søren T. Thomassen, Martin H. Larsen

**Affiliations:** ^1^ Fisheries College Zhejiang Ocean University Zhoushan China; ^2^ Department of Aquatic Resources Institute of Freshwater Research, Swedish University of Agricultural Sciences Drottningholm Sweden; ^3^ Danish Centre for Wild Salmon Randers Denmark; ^4^ National Institute of Aquatic Resources Section for Freshwater Fisheries Ecology, Technical University of Denmark Silkeborg Denmark

**Keywords:** behavioural development, brain size, group rearing, Salmonidae, social isolation

## Abstract

The social environment can affect the development of behavioural phenotypes in fish, and it is important to understand such effects when rearing fish in artificial environments. Here, the authors test the effects of spatial isolation on social interaction propensity and brain development in hatchery‐reared Atlantic salmon *Salmo salar* L. Salmon reared in isolation generally stayed further away from a conspecific in a standardized intruder test than conspecifics reared together in groups. Isolated salmon also tended to be more active in an intruder test, albeit non‐significantly so, but this pattern was not detected in open‐field tests without an intruding conspecific. The cerebellar brain region was relatively smaller in isolated salmon, suggesting that the brain was developing differently in these fish. Therefore, some features of the behavioural and neural phenotype are affected by rearing in isolation. These effects should be considered when rearing salmon, particularly for experimental purposes as it may affect results of laboratory studies on behavioural expression and brain size.

## 1 INTRODUCTION

Conspecifics constitute an integral part of an animal's environment, and the amount and strength of social interactions may lead to adaptive plastic changes in physiology and behaviour, making it possible for individuals to adjust to the presence and competition of the conspecifics (Franck *et al*., 1985; Solomon‐Lane & Hoffmann, 2019). Lack of social stimuli can have detrimental effects in species with high affectionate requirements (Harlow *et al*., 1965; Grippo *et al*., 2007; Christiansen *et al*., 2021), although it may have less effect in solitary or facultatively social species (Stowe *et al*., 2005; Riley *et al*., 2017). Belonging to a social group may also have beneficial effects on cognitive performance, as shown in wild as well as captive‐reared birds (Ashton *et al*., 2018; Langley *et al*., 2018), crabs (Santos *et al*. 2021) and fish (Brandão *et al*., 2015; Ausas *et al*. 2019), but effects are not ubiquitous in the animal kingdom (*e.g*., Riley *et al*. 2018). Overall, studies investigating effects of the social environment on animal individuals can give valuable insights into social requirements for a successful development as a well‐functioning individual.

In fish, long‐term social isolation typically increases aggression in immature individuals, which has been interpreted as an adaptive reaction to obtain and defend territories, and to reach a beneficial social rank in a group of unfamiliar individuals (Franck *et al*., 1985; Hesse & Thünken 2014). Nonetheless, isolation can also suppress the pituitary‐gonadal axis, leading to, *e.g*., reduced aggression in non‐escalating encounters with conspecifics (*e.g*., in cichlids and poeciliids; Hannes & Franck, 1983). Different factors (*e.g*., time of isolation, opponent size or sociability of the species) lead to different adaptive responses to isolation, depending on which type of social interaction has the higher predicted effect on current and future fitness (Franck *et al*., 1985; Gómez‐Laplaza & Morgan, 2000). In addition to its potential effects on aggression, isolation has been shown to reduce both swimming and feeding activity in Arctic charr *Salvelinus alpinus* (L.) juveniles (Leblanc *et al*., 2011, 2019), and foraging rate of novel prey in brown trout *Salmo trutta* L. (Sundström & Johnsson, 2001). Both long‐ and short‐term isolation rearing also reduces general locomotion activity in cichlids like the striped kribensis *Pelvicachromis taeniatus* (Boulenger, 1901) (long‐term isolation) and freshwater angelfish *Pterophyllum scalare* (Schultze, 1823) (short‐term isolation) (Gómez‐Laplaza & Morgan, 1991; Hesse *et al*., 2019). Effects on activity may be species‐specific as no effects were detected in socially isolated zebrafish *Danio rerio* (Hamilton, 1822) (both long‐ and short‐term isolation) (Shams *et al*., 2017). Zebrafish, however, reduce their social preference if isolated in early life, an effect modulated by serotonin (Tunbak *et al*., 2020).

In Atlantic salmon *Salmo salar* L. juveniles, which are typically highly territorial in their natural stream habitat, social interactions are often aggressive (Kalleberg, 1958; Keenleyside & Yamamoto, 1962; Symons & Heland, 1978). Stream‐living salmonids are generally investing substantial amounts of energy into defending resources, in particular against unfamiliar individuals (*e.g*., Taylor, 1988; Höjesjö *et al*., 1998; Závorka *et al*., 2015). Nonetheless, when reared at high densities in aquaculture systems, dominance hierarchies typically collapse (or at least become less polarized) due to general unprofitability of territoriality and aggression in such environments (Yamagishi, 1962; Fernö & Holm, 1986; Metcalfe, 1990). Although the high‐density reared cultured fish have reduced aggression levels at natural densities, they are still more aggressive at high densities than wild salmon (Fenderson & Carpenter, 1971), suggesting that they have not lost the aggressive tendency of the species, but rather show an adaptive response to their environment. Previous experiments investigating experience‐dependent inter‐individual interactions in salmonids have mainly focused on comparing either high‐density hatchery rearing with wild fish or effects of familiarity among individuals (Fenderson & Carpenter, 1971; Olsén *et al*., 1996; Höjesjö *et al*., 1998). Social isolation studies may provide further insights into whether social experience can modify the tendency to interact with conspecifics.

The amount of interactions with conspecifics during ontogeny may also affect the development of the central nervous system, possibly affecting cognitive and behavioural traits, as shown in laboratory‐reared rats (Lipkind *et al*., 2002) and zebra finches (Lapiz et al., 2003; Adar *et al*., 2008). In fish, the environment has been shown to affect brain development, in terms of both gross morphology and cell proliferation (Ebbesson & Braithwaite, 2012; Johnsson *et al*., 2014; Dunlap, 2016; Ausas *et al*., 2019). Changes in brain morphology can appear within as little as 2 weeks, with associated changes in behaviour (Joyce & Brown, 2020a,b). The brain growth and development of fishes have also specifically been shown to respond to the number of conspecifics in the rearing environment (Fischer *et al*., 2015; Näslund *et al*., 2017b, 2019), as well as social isolation *per se* (Coss & Globus, 1979; Gonda *et al*., 2009; Dunlap *et al*., 2011).

In this study, the authors compare the social behaviour and brain size of isolation‐reared and group‐reared Atlantic salmon from a conservation hatchery during the initial life stage. Although isolation‐rearing is not likely a feasible method for high production hatchery facilities, this extreme‐case set‐up can provide insights into potential differences stemming from density manipulations and a reference for comparisons with other rearing density levels. More importantly, isolation‐rearing is commonly used in basic research projects on fish, and the results will thereby inform on potential environmental effects of social isolation on salmon behaviour and brain size development, a necessity for correct interpretation of results from such studies.

Given previous effects of rearing density on the brain size of Atlantic salmon, repeatedly showing a positive relationship between cerebellum size and fish density (Näslund *et al*., 2017b, 2019), the authors expected similar effects in this study such that isolation‐reared fish should develop relatively smaller cerebellum than group‐reared individuals. Other brain regions were investigated from an exploratory perspective to obtain an overview of potential trade‐offs among these regions, given that no overall difference in total brain size has been indicated for Atlantic salmon in different rearing environments (Näslund *et al*., 2017b). With respect to behaviour, the authors hypothesized that salmon without any prior experience of other conspecifics would show either increased interaction propensity due to higher aggression, as suggested for most social isolation studies on juvenile fish (Franck *et al*., 1985), or decreased interaction propensity if the situation would be experienced as potentially risky for the isolated individual. Data were also explored, without prior hypothesizing, with respect to relationships between brain region size and behavioural expression.

## 2 MATERIALS AND METHODS

### 2.1 Rearing conditions

**FIGURE 1:**
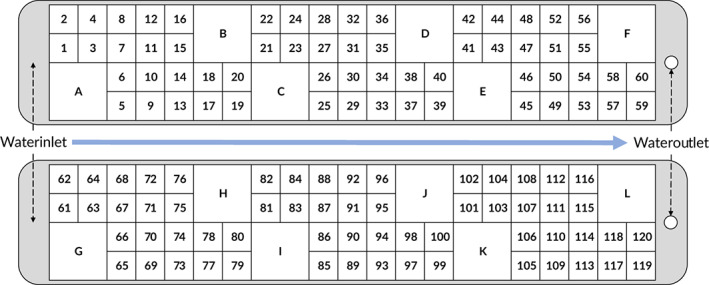
Layout of the rearing compartments in the two experimental runways. Isolation‐reared fish were reared in numbered compartments (1–120); group‐reared fish were reared in lettered compartments (A–L), with 300 individuals in each group (a sub‐set, *n* = 36, was analysed for brain‐ and behavioural differences). Water flowed through all compartments from the inlet to the outlet

Fertilized eggs from wild Atlantic salmon, caught by electrofishing (River Gudenaa, Denmark, late autumn 2015), were incubated in hatching trays and hatched in late March 2016. On 13 May 2016, at the start of exogenous feeding after yolk‐sac absorption, fish were randomly collected from the hatching trays and transferred to two experimental runways (40 × 360 cm). Each runway was divided into 60 small (10 × 15 cm) and 6 large (20 × 30 cm) compartments by perforated plastic mesh screens (Figure 1). A single fish was reared in each of the small compartments (hereafter referred to as “isolation‐reared”), and 300 individuals were reared in each of the large compartments (hereafter referred to as “group‐reared”). The diameter of the mesh holes was 1 mm, reducing but not eliminating visual and chemical contact between isolated individuals. The experimental design assumes that available water volume per individual is not the driving factor for any induced effects.

The runways were supplied with water from a recirculating system at a flow rate of *c*. 20 l min^−1^. The water depth was 15 cm, and the average water temperature during the rearing period was 13.1°C (range: 10.3–15.0°C). Fish were hand‐fed with dry feed pellets six times per day (06.00, 09.00, 12.00, 15.00, 18.00, 21.00 hours) in all compartments and given a daily ration representing 2%–4% of body mass.

On 14 May, a sub‐sample of 60 fish from each of the two rearing treatments (*i.e*., isolation‐reared and group‐reared) was randomly collected using a net in the experimental runways to determine initial total length and body mass to the nearest 0.1 mm and 0.01 g, respectively. The fish were returned to their respective compartments after these measurements were made.

### 2.2 Open‐field and intruder tests

Between 24 and 26 July 2016, the individual behaviour of 36 fish from each rearing treatment was scored in an open‐field and an intruder test. For these behavioural tests, three group‐reared fish were randomly collected from each of the 12 large compartments, whereas 18 isolation‐reared fish were sampled at random from each runway.

The behavioural tests were conducted in four opaque white plastic tanks (38.5 × 27.0 cm; water level: 5 cm), positioned underneath a digital single‐lens reflex (DSLR) camera (Canon EOS 60D; Canon Inc., Tokyo, Japan) mounted with a wide‐angle lens (Samyang 24 mm f/1.4; Samyang Optics Co., Ltd., Masan, South Korea) to allow for simultaneous recording. Lens distortion was considered negligible and was not adjusted for (see Supporting Information Figure S1, Appendix S1). The entire experimental set‐up was surrounded by an observation blind constructed from green tarpaulin to ensure that fish were not disturbed during behavioural scoring. The behavioural arenas were filled with water acquired from the main recirculating system and were changed between each behavioural trial round (*N* = 18). The average water temperature during the behavioural tests was 13.7°C (range: 13.5–14.0°C), and the light intensity was 120 lx. Behavioural trials were conducted over three consecutive days, between 9.00 and 17.00 hours.

In the open‐field test, a single fish was placed in each of the four behavioural arenas. Two randomly chosen fish from each rearing treatment were scored in each trial round to minimize possible time effects. Immediately after the fish was introduced to the arena, the movement rate (MR, cm s^−1^) of the salmon was video recorded for 20 min. Ten minutes after completion of the open‐field test, an intruder fish was placed into a small transparent glass jar (Ø = 8 cm, height = 10 cm, water level = 5 cm), positioned at the centre of the behavioural arenas. The intruder fish were size‐matched with the focal fish (max 14% length difference), as aggression is typically strongest between similar‐sized fish (Guo et al., 2017), and collected from a separate rearing unit that was not part of this study. The behaviour of the focal fish was video recorded for 20 min to measure the MR (cm∙s^−1^) and the distance between the focal and intruder fish (cm).

### 2.3 Animal tracking

Automated animal tracking from recorded videos was conducted using the idTracker software (Pérez‐Escudero *et al*., 2014). For the open‐field trials, videos were recorded for 15 min and were analysed after discarding the first minute after the fish were introduced into the arenas, to avoid scoring the initial behavioural responses of the fish to the new environment. For intruder trials, videos were scored for 12 min after discarding the first minute after the introduction of the intruder individual. The discrepancy in tracking time between trials was due to malfunction of one video‐recording after 13 min in the intruder test.

Output tracks from idTracker, based on *X*‐ and *Y*‐coordinates from the recorded videos, were smoothed using a rolling mean function (window size = 15 frames), using the R‐package TTR (Ulrich, 2018). Distance moved was obtained using the R‐package adehabitatLT (Calenge, 2016) and converted to mean MR by dividing the total distance moved by the time‐length of the tracked video segment. Inter‐individual distance between focal individuals and intruders was calculated for each video frame as the Euclidean distance between the coordinate‐pairs for the respective individual and averaged over the length of the track. Tracking and track‐calculations were conducted blindly with respect to rearing treatment.

### 2.4 Brain measurements

**FIGURE 2:**
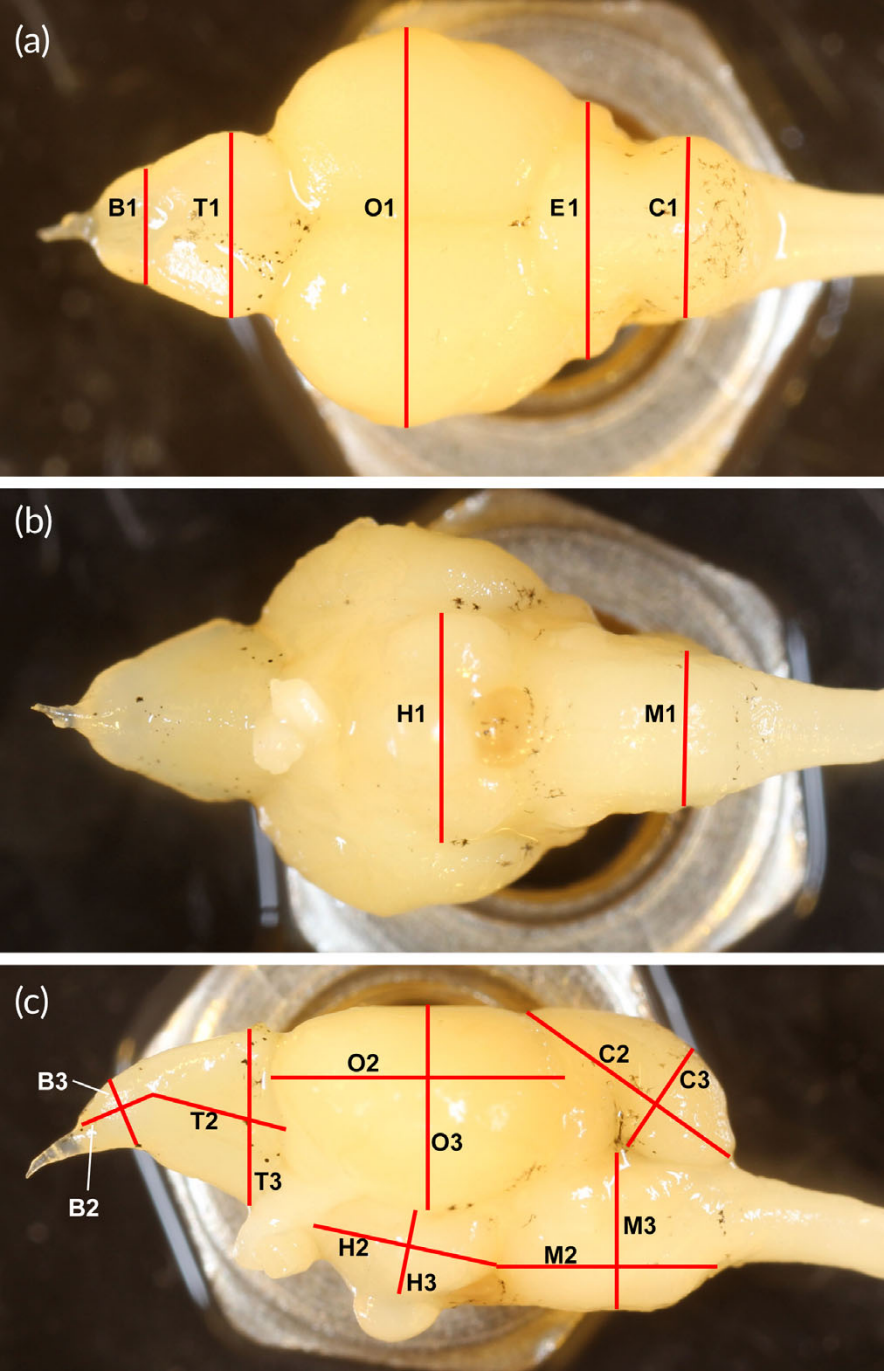
Brain measurements. (a) Dorsal measurements, (b) ventral measurements, (c) lateral measurements. Key to measurements: B – olfactory bulbs, T – telencephalon, O – optic tectum, C – corpus cerebellum, E – eminentia granularis, H – hypothalamus, M – medulla oblongata

After behavioural scoring, the focal individuals were killed by an overdose of benzocaine and decapitated. Body length and mass of the fish were measured as mentioned earlier. Heads were stored in 4% phosphate‐buffered formaldehyde at 10°C until dissection. Brains were dissected under light‐microscope, and photographed dorsally, ventrally and laterally using a DSLR camera (Canon EOS 40D, Canon Inc.) with a mounted super‐macro lens (Canon EF MP‐E65 f/2.8 1‐5X, Canon Inc.). During photography, the brains were immersed in phosphate‐buffered saline solution and placed on a hardware nut (visible in Figure 2) to facilitate appropriate perpendicular aligning to the camera lens. From the photographs, 19 linear metrics were taken, as described in Figure 2, using the software ImageJ (Schneider *et al*., 2012). Dissections and measurements were conducted blindly with respect to treatment.

### 2.5 Statistical analyses: general information

All statistical analyses were conducted in R 3.4.2 (R Core Team, 2022). Graphs were constructed using ggplot2 (Wickham, 2011) and cowplot (Wilke, 2020).

### 2.6 Statistical analyses for fish size and behavioural data

Body size (wet mass and total length) at the start of the experiment (*i.e*., at start‐feeding) was compared using a linear model, with treatment as the only factor. Body size at the time of behavioural trials was analysed in the same way. The effects of fish body size on MR (cm∙s^−1^) and inter‐individual distance (cm), which may be present in juvenile salmonids (*e.g*., Näslund *et al*., 2017a; Lovén Wallerius *et al*., 2017), were initially investigated for each treatment group (*i.e*., isolation‐reared and group‐reared) by linear regressions. These regressions indicated no strong directional effects of body size on either MR or inter‐individual distance (all *R* ^2^ < 0.06, all *P* > 0.17). Effects of trial day on MR and inter‐individual distance were also found to be negligible based on graphical examination of box plots. Effects of rearing compartment for group‐reared fish were not considered for any analyses. Differences among tanks were initially analysed separately for all analysed variables, using linear models with tank ID and body length as independent factors, and no strong evidence was found for tank‐related differences (all *P* > 0.09). Graphical investigation revealed no additional concerns in cases with *P* < 0.1, as no individual tank had data systematically different from other tanks. Normality and homoscedasticity were assessed graphically, using box plot symmetry and spread and histograms of model residuals (Supporting Information Figure S2 in Appendix S1).

**FIGURE 3:**
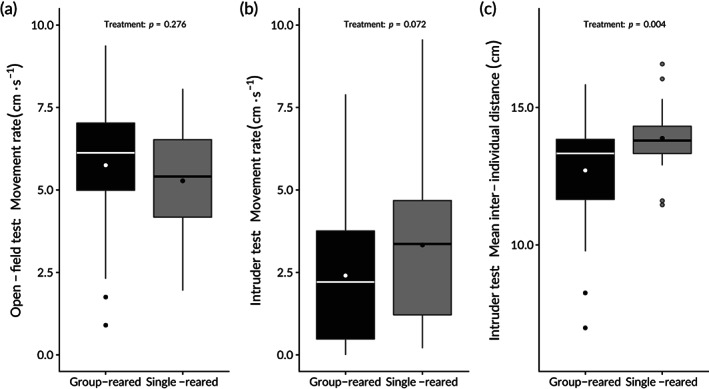
Box‐and‐whisker plot showing the behaviour of isolation‐reared and group‐reared Atlantic salmon in the behavioural trials. (a) Movement rate in open‐field trials. (b) Movement rate in intruder trials. (c) Inter‐individual distance between focal individual and stimuli intruder. Box‐hinges mark the first and third quartiles, with median drawn as a horizontal bar across the box and mean depicted with a dot within the box. Whiskers extend to the farthest data point within 2/3 interquartile range from a box‐hinge; points farther away are depicted as dots

Movement rates of isolation‐reared and group‐reared salmon in the open‐field and intruder test were compared using two‐sided Welch's *t*‐tests, allowing for heterogeneous variances. MR in the intruder test was log_e_‐transformed, due to positively skewed box plots (see Figure 3b). The same statistical procedure was applied to compare the inter‐individual distance of isolation‐reared and group‐reared salmon with the intruder fish (analysed untransformed).

Spearman's rank correlations were applied to investigate relationships between (a) the activity of the focal individual and the intruder, and (b) the activity of the focal fish between the two behavioural tests. Change in activity of the focal fish from the open‐field test to the intruder test was analysed using a paired *t*‐test.

### 2.7 Statistical analyses for brain data

Missing data for brain subregion measurements (*N*_B1_ = 9; *N*_B2_ = 5; *N*_T1_ = 1; *N*_T2_ = 1; *N*_E1_ = 1; *N*_H2_ = 2; *N*_H3_ = 2; all other = 0), due to damage during dissections, were imputed using the probabilistic principal component (ppca) method in the pcaMethods package for *R* (Stacklies & Redestig, 2017; Stacklies *et al*., 2007). All 19 linear measurements were added as variables within the ppca procedure. Imputation was necessary to allow for total brain volume to be used as a covariate in linear models of brain subregion size (see below).

Volumes (*V*) of brain subregions were calculated based on the idealized ellipsoid model (Huber *et al*., 1997):

$$V=\left(W∙L∙H\right)\pi /6$$

where *W* is the width of the subregion, *L* is the length and *H* is the height. For subregions with two lobes (olfactory bulbs, telencephalon, optic tectum and hypothalamus), the width was divided by 2 within the formula, and the resulting volume estimate was multiplied by 2 to get the volume estimate of the two lobes combined. Although it is known that the ellipsoid model is not estimating perfect subregion volumes in absolute terms (Pollen *et al*., 2007; Ullmann *et al*., 2010), it still represents a combination of the *W*, *L* and *H* measurements, which are measured with high precision (the π/6 term is just a transformation to get a value roughly representing the real volume). For total brain volume was estimated by adding all subregions together. The eminentia granularis (E1) measurement was used only within the imputation procedure, not in volume calculations.

For the analysis of total brain volume, a linear model was used with log_e_(volume) as the response variable, total length of the fish as a covariate and treatment added as a fixed factor. For each subregion, a separate model was run with log_e_(volume) as the response variable, total brain volume minus the analysed subregion volume as a covariate and treatment added as a fixed factor.

Separate linear models were used to explore potential relationships of brain‐ and region size to the MRs in the behavioural tests. For the MR in the open‐field test the authors included brain/region size (log_e_‐transformed) as the independent focal variable, and either body length (together with total brain size) or log_e_(total brain volume) (together with brain region size) as covariates to correct for size effects. For the MR in the intruder test the authors also included the intruder MR as an additional covariate.

In all models, they assumed that rearing compartments for the group‐reared fish provided identical effects on the brain measurements.

## 3 RESULTS

### 3.1 Size and mortality

The average total body length and wet mass at start‐feeding did not differ significantly between the two rearing groups (length: *F*_1,118_ = 0.117, *P* = 0.734; body mass: *F*_1,118_ = 3.032, *P* = 0.084). No significant size differences were found at the time of trials either (length: *F*_1,70_ = 0.064, *P* = 0.937; body mass: *F*_1,70_ = 3.070, *P* = 0.084). The overall mortality rate during rearing was 15% for isolation‐reared fish and 9% for group‐reared fish (*χ* ^2^‐test: *P* = 0.022), which was deemed comparable to that experienced by other salmon in the hatchery, not a part of this study (MHL, pers. obs.).

### 3.2 Behaviour in open‐field and intruder tests

In the open‐field test, no effect of rearing treatment was found on MR [|*t*| = 1.099, *df* = 67.20, *P* = 0.276; 95% c.i. for estimated difference (group‐reared mean – isolation‐reared mean): −0.39 – 1.33 cm∙s^−1^; Figure 3a]. In the intruder test, a non‐significant trend towards isolation‐reared fish being more active than group‐reared fish was found [|*t*| = 1.77, *df* = 68.64, *P* = 0.08; 95% c.i. for estimated difference (group‐reared mean – isolation‐reared mean): −1.98 – 0.11 cm∙s^−1^; Figure 3b].

In the intruder test, isolation‐reared fish on average maintained a larger distance between themselves and the intruder individual than group‐reared fish [|*t*| = 3.03, *df* = 51.07, *P* = 0.004; 95% c.i. for estimated difference (group‐reared mean – isolation‐reared mean): −1.94 – −0.40 cm∙s^−1^; Figure 3c]. Spearman's rank correlation suggested a positive, but non‐significant, trend correlation between the movement activity between focal individuals and intruders (*ρ* = 0.21, *P* = 0.08).

In general, fish decreased their MR from the open‐field test to the intruder test [|*t*| = 8.17, *df* = 71, *P* < 0.001; mean difference ± 95% c.i. (2.65 ± 0.65); Supporting Information Figure S3 in Appendix S1]. Nonetheless, no appreciable rank correlation was found for movement activity in both the open‐field and intruder test (*ρ* = 0.15, *P* = 0.20).

### 3.3 Brain size

**FIGURE 4:**
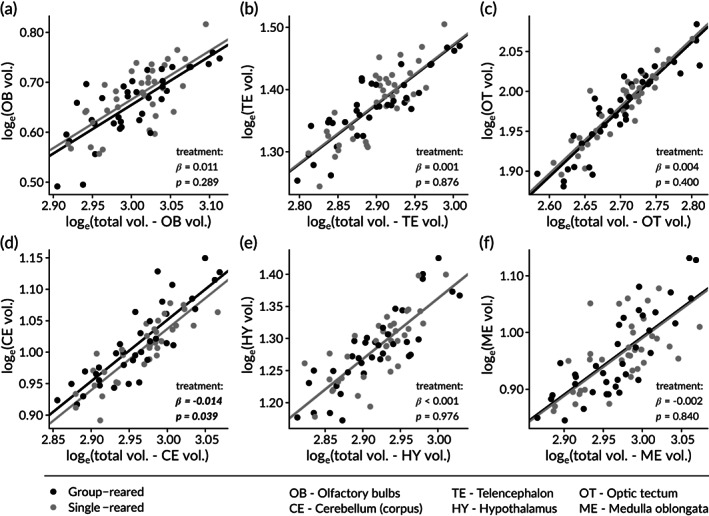
Calculated volume of brain subregions: (a) olfactory bulbs, (b) telencephalon, (c) optic tectum, (d) cerebellum, (e) hypothalamus, and (f) medulla oblongata. *Y*‐axes represent the log_e_‐transformed volume of the respective subregion, and *X*‐axes represent the log_e_‐transformed whole brain volume minus the analysed brain region. Black lines show the regression for group‐reared fish, and the grey line show the regression for the isolation‐reared fish; *β*‐values represent the estimated difference between group‐ and isolation‐reared fish, and the *P*‐value indicates the statistical significance. Differences between treatments were significant only for cerebellum, panel d

Total brain size was not markedly affected by rearing treatment (Table 1). Neither was any subregion, except cerebellum, affected by the treatment (Table 1; Figure 4). Cerebellum was smaller on average in salmon reared in isolation as compared to group‐reared salmon (Table 1; Figure 4d). Covariates (whole brain volume: total length in millimetre; substructure volumes: total brain volume minus the analysed substructure itself) had a strong positive influence in all analyses (Table 1).

**TABLE 1:** Summary table for all analyses concerning total brain volume and subregion volumes

Analysed volume	Coefficient	Estimate	Std. error	*t*‐value	*P*‐value
log_e_(whole brain, “TOT”)	Intercept	2.635	0.023	115.4	**<0.001**
	Total length	0.008	<0.001	20.27	**<0.001**
	Treatment	0.003	0.004	0.702	0.485
log_e_(olfactory bulbs, “OB”)	Intercept	−2.262	0.315	−7.177	**<0.001**
	log_e_(TOT – OB)	0.972	0.105	9.264	**<0.001**
	Treatment	0.011	0.010	1.068	0.289
log_e_(telencephalon, “TE”)	Intercept	−1.388	0.223	−6.229	**<0.001**
	log_e_(TOT – TE)	0.953	0.077	12.39	**<0.001**
	Treatment	0.001	0.007	0.157	0.876
log_e_(optic tectum, “OT”)	Intercept	−0.301	0.123	−2.448	**0.017**
	log_e_(TOT – OT)	0.844	0.046	18.53	**<0.001**
	Treatment	0.004	0.005	0.846	0.400
log_e_(cerebellum, “CE”)	Intercept	−1.873	0.206	−9.095	**<0.001**
	log_e_(TOT – CE)	0.975	0.070	14.02	**<0.001**
	Treatment	−0.014	0.007	−2.101	**0.039**
log_e_(hypothalamus, “HY”)	Intercept	−1.475	0.234	−6.308	**<0.001**
	log_e_(TOT – HY)	0.946	0.080	11.80	**<0.001**
	Treatment	<0.001	0.008	0.030	0.979
log_e_(medulla oblongata, “ME”)	Intercept	−2.062	0.338	−6.094	**<0.001**
	log_e_(TOT – ME)	1.018	0.114	8.933	**<0.001**
	Treatment	−0.002	0.011	−0.202	0.840

*Note*: Estimates for the treatment coefficient relate to how much isolation‐reared fish differ from group‐reared fish. All models were significantly different from the intercept‐only model (*F*_2,69_ > 39; *P* < 0.001 in all cases). Bold *P*‐values indicate statistical significance (*P* < 0.05).

### 3.4 Correlations between brain size and behaviour

No significant relationships were found between MR in the open‐field test and any of the size‐corrected brain size or brain region size estimates (all *P* > 0.11). Similarly, no significant associations were found between MR and brain size in the intruder test (all *P* > 0.08). For the latter suite of analyses, the lowest *P*‐value (0.08, relating to the analysis of movement as dependent on relative cerebellar size) is relatively close to the significance threshold (*P* > 0.21 for all other analyses). The parameter estimate (*β* = −6.6, s.e.: 3.8) suggests a possible negative relationship between cerebellar size and MR in the intruder test, but given the multiple testing performed the authors only make a note about this possibly interesting pattern and refrain from further discussions about potential biological significance; follow‐up studies could be warranted.

## 4 DISCUSSION

From this study, the authors present two main results: (a) that isolation‐rearing of juvenile Atlantic salmon reduces the average interaction propensity of the isolated individuals with a conspecific and (b) that isolation‐rearing leads to a reduction in the size of the cerebellar region of the brain as compared with fish reared in groups. In the standard open‐field trials there appear to be no major effects of isolation in this species, in contrast with some other species (Gómez‐Laplaza & Morgan, 1991; Hesse *et al*., 2019).

### 4.1 Decreased willingness to associate with conspecifics in isolated salmon

Social interactions during early life have previously been shown to improve the ability to adopt adequate social behaviours in dominance hierarchies in daffodil cichlids *Neolamprologus pulcher* (Trewavas & Poll, 1952) (Fischer *et al*., 2015). Visual attraction to conspecifics has been shown to be innate in the naturally shoaling medaka *Oryzias latipes* (Temminck & Schlegel, 1846), with no effect of social isolation on this behaviour (Aoki, 1980). Although salmonid juveniles of the genus *Salmo* are typically territorial, they still commonly encounter conspecifics in the wild and benefit from assessing their place in social hierarchies in relation to their own competitive capacity (Jenkins, 1969; Griffiths *et al*., 2004). The results suggest that isolation from other conspecifics may decrease their willingness to interact with competitors, at least in the short term. The possibility that rearing conditions (group‐ or isolation‐rearing) can affect social or agonistic interactions in salmonids needs to be considered when comparing studies investigating these traits, particularly when estimating effects sizes across multiple studies.

Other studies on different species have also found effects on social interactions stemming from isolation, mainly in terms of aggressive behaviour. A study on adult Siamese fighting fish *Betta splendens* Regan, 1910 showed that males isolated for a few weeks decreased their readiness to perform aggressive display towards a model, but increased the display rates once they had been primed with an aggression‐provoking stimulus (Halperin *et al*., 1992). Short‐term isolation in adult paradise fish *Macropodus opercularis* (L.) leads to increased aggression (Davis et al., 1974). Nonetheless, these adult paradise fish had previous experience of direct contact with conspecifics. Although fish in this study had some previous experience of group rearing during the yolk‐sac stage, little aggression is seen during this stage in Atlantic salmon (Dill, 1977), and the social and aggressive experience is thereby likely more limited than for the paradise fish. Furthermore, aggression did not seem to be the main driver behind the effects in this study (discussed later). Other behavioural effects relating to inter‐individual interactions include aberrant schooling behaviour, including collisions between individuals, in *Menidia* sp. fry reared in isolation (Shaw, 1960), which may indicate that body coordination had been affected.

In the present experiment, the authors assumed that aggression would be a driving factor determining inter‐individual distance patterns in the intruder test. Nonetheless, salmon exposed to the intruder did not show overt aggression as often seen in young salmonids confronted with mirrors (*e.g*., Holtby *et al*., 1993; Näslund & Johnsson, 2016), and threat behaviours (Keenleyside & Yamamoto, 1962) could not be confidently determined from the videos. Rather, fish interacting with the intruder tended to spend part of the time swimming around the glass jar holding the intruder, and the rest of the time without directed movements towards the intruder. This does not exclude aggressive motivation for the approaches, but may be an effect of the lack of direct contact with the intruder. Nonetheless, most individuals did not interact much with the intruder, suggesting that the effects seen between the treatments were more due to avoidance behaviours, rather than aggression. Examples of videos from the intruder test are uploaded to the figshare database (https://doi.org/10.6084/m9.figshare.14527242).

Possibly, the detected effect in the intruder test could possibly be modulated by serotonin expression, which is associated to social interaction behaviours and has been shown to be modified by social isolation (Winberg & Thörnqvist, 2016; Backström & Winberg, 2017; Otsuka *et al*., 2020; Tunbak *et al*., 2020).

The authors evaluated behavioural effects on fish coming directly from an isolation treatment. Therefore, it is possible that the fish could plastically modify their response to conspecifics after a time of direct‐contact experience with conspecifics. The main implication of the results is that isolated Atlantic salmon may behave differently towards conspecifics than group‐reared individuals in, for example, experimental trials involving social interactions.

### 4.2 Reduced cerebellar size in isolated salmon

When investigating the relative size of the cerebellum in relation to the whole brain size, the authors found that isolation‐reared fish had smaller cerebella than group‐reared fish. No effects were found on any other brain regions. The observed effect is consistent with previous studies investigating effects of fish density on the brain in Atlantic salmon (Näslund *et al*., 2017b, 2019) and daffodil cichlids (Fischer *et al*., 2015). The previous salmon studies have compared the brain of fish reared at different stocking densities (1500 *vs*. 500 individuals∙m^−2^ in Näslund *et al*., 2017b; 150 *vs*. 50 individuals∙m^−2^ in Näslund *et al*., 2019). The consistency in the effect pattern suggests robust effect patterns with respect to more conspecifics in the surrounding environment leading to larger cerebella. The fish used in the present study were smaller and younger than the fish in the previous experiments, and thus had less time to develop the observed differences in cerebellar size. This could potentially explain the fact that the effect is rather small. The cause of the differentiation in the cerebellar region cannot be determined in the present study, and nor in the previous studies (Näslund *et al*., 2017b, 2019). Studies on three‐spined sticklebacks *Gasterosteus aculeatus* L. have shown that socially housed females differ from isolated females in their brain gene‐expression, particularly in the cerebellum, with upregulated genes in the social group including several genes related to neural development in the hindbrain (Greenwood & Peichel, 2015).

Because the cerebellum has a role in the coordination of movements (Yopak *et al*. 2016), the authors continue to promote the previously posed hypothesis that environments with higher numbers of individuals lead to a stimulatory effect on cerebellar growth due to increased demand for motor performance and manoeuvrability (Näslund *et al*., 2017b, 2019). They also acknowledge the need for further experimentation to uncover the mechanisms behind this pattern. The cerebellar structure is proposed to have a wide variety of functions, including being an associative centre for sensory input and involved in several cognitive functions (Yopak *et al*., 2016), and thus there might be other explanations for its response to social‐ and density treatments. Overall, the social/density‐related effects on cerebellum appear to be a good and robust candidate system for further investigations on the mechanisms underlying brain plasticity in salmonids.

The detected effects on the brain differ from effects of isolation on the brain of nine‐spined sticklebacks *Pungitius pungitius* (L.) where decreased size of optic tectum and olfactory bulbs was found (Gonda *et al*., 2009). Local density of conspecifics has also been shown positively associated with the size of the forebrain (telencephalon and diencephalon) in wild bluestreak cleaner wrasse *Labroides dimidiatus* (Valenciennes, 1839) (Triki *et al*., 2019). In rainbow trout *Oncorhynchus mykiss* (Walbaum, 1792), social isolation for 4 weeks reduced neurogenesis in the dorsomedial telencephalon (Ausas *et al*., 2019), but consequent changes in overall size of telencephalon are possibly not necessarily seen. Discrepancy in brain subregions affected by social environment points towards species‐specific responses and suggests that inter‐specific inference is not advisable in studies of environmental effects on fish brains.

### 4.3 Study caveats

The two rearing groups had slightly different mortality rates (isolation‐reared fish having a higher mortality rate), which may generate differences in the surviving individuals, should the mortality be due to a selective agent. The authors deem the mortality rates to be reasonable for salmon of the age used, given that some fish are of lower vitality in hatcheries where no natural selection is acting on the initial stages.

To standardize the test of social interaction propensity, the authors chose to confine a size‐matched individual in a round container in the trial arena. Although this procedure was the same for both treatments, the confinement of the intruder individual may cause an unnatural behaviour. Nevertheless, the different treatment led to different reactions in the focal fish, showing that they approach conspecifics in different ways. Whether the approach is driven by aggression or curiosity‐like behaviour, or a combination of these, is impossible to determine from the present analysis. Furthermore, there were no non‐intruder controls introducing only an empty glass jar. This makes it impossible to distinguish intruder effects from novel object effects. Nonetheless, other studies in salmonids show that conspecific intruder stimuli are very strong (*e.g*., Keenleyside & Yamamoto 1962; Näslund & Johnsson, 2016; Johnsson & Näslund, 2018), and therefore, it is assumed that the intruder individual has higher valence as a stimulus than the glass jar in this experimental set‐up. Further studies utilizing other social interaction and aggression protocols, *e.g*., dyads and mirror‐image stimulation, may elucidate these questions.

## 5 CONCLUSIONS

The results of the present study indicate effects of social environment on behaviour and cerebellar growth in Atlantic salmon. Spatial isolation led to a lowered willingness to approach conspecifics and a smaller cerebellar brain region. The results highlight the importance of considering the housing conditions in laboratory experiments.

## AUTHOR CONTRIBUTIONS

M.H.L. conceived and conducted the main experiment. J.N. and M.H.L. conceived and designed the behavioural trials. H.G. performed brain dissections and measurements. J.N. analysed the data and wrote the initial draft of the manuscript, with input from M.H.L. H.G. and S.T.T. contributed through discussions and advice regarding experimental design, interpretation of data and manuscript content.

## CONFLICT OF INTEREST

The authors declare no conflicts of interest.

## ETHICS STATEMENT

All applicable international and national guidelines for the care and use of animals were followed. The experiment was conducted in accordance to the guidelines described in permission 2012‐DY‐2934‐00007 from the Danish Experimental Animal Committee.

## Supporting information


**APPENDIX S1** Supporting information

## Data Availability

Data and R‐code used in analyses are accessible in the figshare database: https://doi.org/10.6084/m9.figshare.14527242.
