# Supplementary material for: Social isolation affects intra‐specific interaction behaviour and reduces the size of the cerebellar brain region in juvenile Atlantic salmon Salmo salar
Source: J Fish Biol. 2022 Jul 13;101(3):711–21. doi: 10.1111/jfb.15142 (PMC9540882; doi:10.1111/jfb.15142)
Supplement: Supplementary file 1 — APPENDIX S1 Supporting information [file JFB-101-711-s001.docx]

**SUPPLEMENTARY MATERIALS**

**Social isolation affects intra-specific interaction behaviour and reduces the size of the cerebellar brain region in juvenile Atlantic salmon *Salmo salar***

Guo H^a^, Näslund J^b^ , Thomassen ST^c^, Larsen MH^c^

1. **Fisheries College, Zhejiang Ocean University, Zhoushan, China**
2. **Department of Aquatic Resources, Institute of Freshwater Research, Swedish University of Agricultural Sciences**
3. **Danish Centre for Wild Salmon, Randers, Denmark**
4. **National Institute of Aquatic Resources, Section for Freshwater Fisheries Ecology, Technical University of Denmark**

*** Corresponding authors:**

**Joacim Näslund: joacim.naslund@slu.se**

**Martin H. Larsen: mhl@vildlaks.dk**


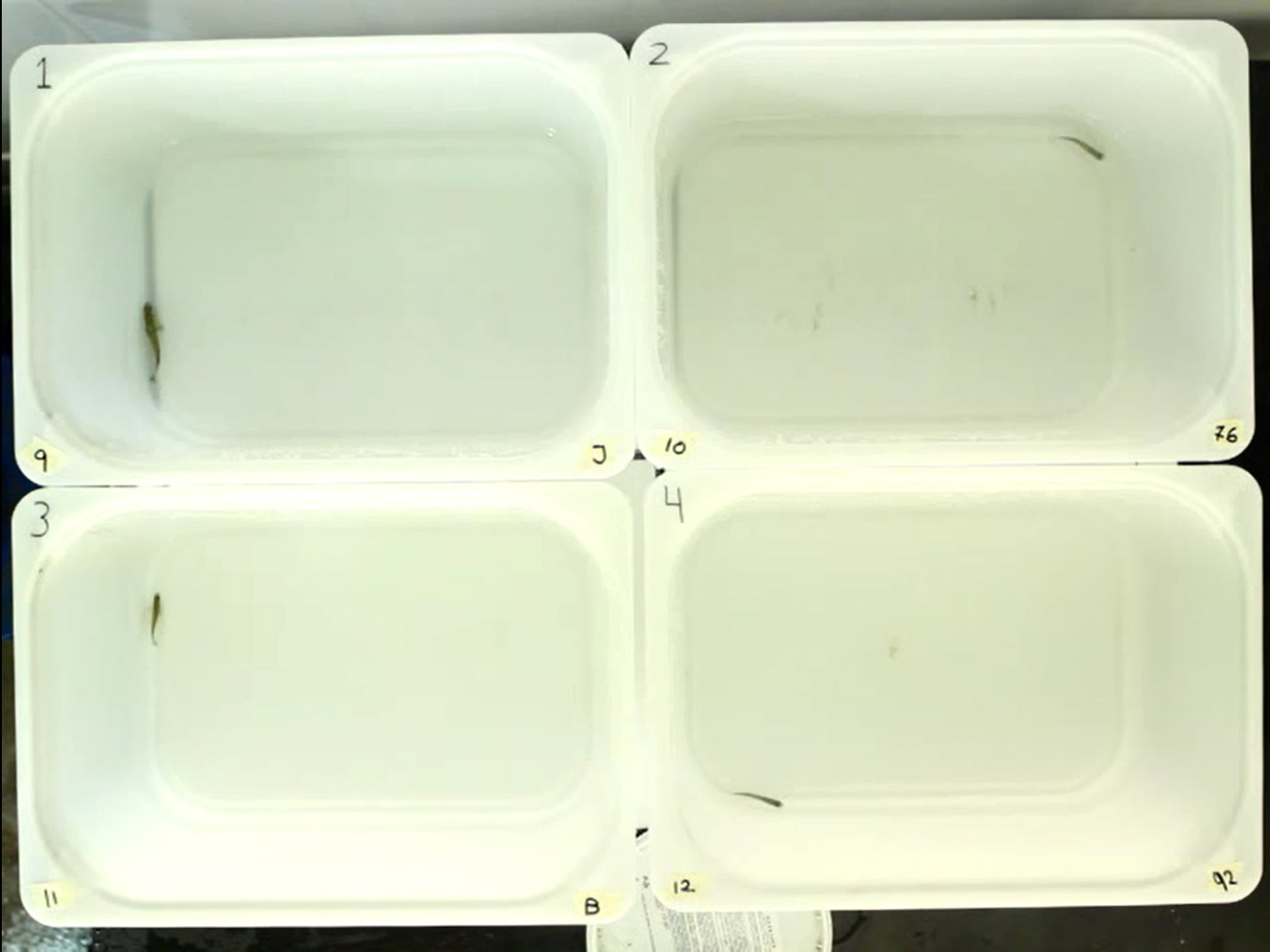


**Figure S1.** Unedited screenshot from a recording of the open-field trials.


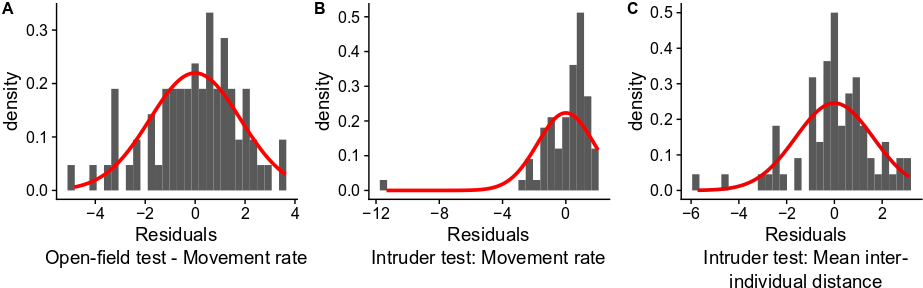


**Figure S2.** Normal density curves and histograms of model residuals for A) open-field movement rate, B) intruder test movement rate, and C) intruder test inter-individual distance. See Figure 3 in the main text for box-plots of the raw data.

**Figure S3.** Relationship between activity in the open-field test and intruder test. The red line depicts the 1:1 relationship; points below this line indicate reduced activity in the Intruder test, as compared to the open-field test.
